# AI-Based Gait Analysis System for Rehabilitation: Usability Evaluation of Human-Technology Interaction

**DOI:** 10.2196/80748

**Published:** 2025-12-18

**Authors:** Seojin Hong, Hyun Choi, Hyosun Kweon

**Affiliations:** 1 Department of Clinical Rehabilitation Research Rehabilitation Research Institute National Rehabilitation Center Seoul Republic of Korea; 2 Department of Healthcare and Public Health Rehabilitation Research Institute National Rehabilitation Center Seoul Republic of Korea

**Keywords:** usability evaluation, AI-based gait analysis, focus group interview, system usability scale, rehabilitation

## Abstract

**Background:**

Artificial intelligence (AI)–based gait analysis systems are increasingly applied in rehabilitation settings for objective and quantitative assessment of gait function. However, despite their potential, clinical adoption remains limited due to insufficient consideration of usability, user experience, and integration into actual clinical workflows.

**Objective:**

This study aimed to conduct a formative evaluation of a prototype AI-based gait analysis system (MediStep M).

**Methods:**

A mixed methods formative usability evaluation was conducted with 5 licensed physical therapists. Qualitative data were collected through focus group interviews, and quantitative usability was measured using the system usability scale (SUS). A scenario-based usability assessment was applied to identify user interface challenges, workflow issues, and potential design improvements.

**Results:**

Participants identified major usability barriers, including limited accessibility of the power button, absence of battery status indicators, burdensome manual calibration, and insufficient clinical detail in the gait analysis reports. They also emphasized the need for wireless operation, improved portability, and integration with hospital electronic medical record systems. The mean SUS score was 57 (grade D), indicating suboptimal usability and the need for iterative design refinements.

**Conclusions:**

Although AI-based gait analysis systems hold promise for enhancing rehabilitation outcomes, key usability challenges must be resolved before clinical implementation. Improvements in hardware portability, automated calibration, data management, and user interface design are essential to ensure safety, efficiency, and clinical applicability. These findings provide evidence-based insights to guide iterative development and promote user-centered innovation in AI-based rehabilitation technologies.

## Introduction

Gait analysis plays a critical role in the rehabilitation of individuals with neurological or musculoskeletal impairments [1-4]. Quantitative assessment of gait function enables clinicians to objectively evaluate motor performance, establish evidence-based treatment plans, and monitor functional recovery over time [2,5]. However, conventional gait analysis systems such as treadmill-based or marker-dependent motion capture platforms require specialized laboratories, trained operators, and controlled environments [6,7]. These limitations restrict their feasibility for routine clinical application and hinder broader accessibility in diverse rehabilitation settings [6,8].

Recent advancements in artificial intelligence (AI), computer vision, and depth-sensing technologies have facilitated the development of markerless, AI-driven gait analysis systems capable of automatically estimating joint coordinates and computing spatiotemporal gait parameters [9-12]. Such systems have the potential to make gait analysis more portable, cost-effective, and clinically scalable [6,10,13]. Compared to conventional systems such as Zebris, which rely on treadmill-based assessments within constrained spatial setups, emerging overground AI-based systems are designed to enable natural walking conditions with minimal setup requirements [9,14].

Despite these technological advancements, many AI-based gait analysis systems continue to face significant usability challenges that limit their integration into real-world rehabilitation environments. Reported issues include labor-intensive calibration procedures, unclear user feedback interfaces, hardware nonportability, and poor alignment with clinical workflows. Previous research in human factors and digital rehabilitation has emphasized that usability—particularly intuitiveness of the user interface, workflow efficiency, and error prevention—is a primary determinant of successful clinical adoption and user satisfaction [15].

To address these challenges, we conducted a formative evaluation of a prototype AI-based gait analysis system, MediStep M. The evaluation was performed with physical therapists, who represent the primary end users of gait analysis technologies in clinical practice. A mixed methods approach was employed, combining qualitative focus group interviews (FGIs) with quantitative assessments using the system usability scale (SUS). This approach aimed to identify use-related problems, interface limitations, and potential design improvements to guide iterative enhancement of AI-based rehabilitation technologies and to ensure their alignment with clinical usability requirements.

## Methods

### Study Design

This study employed a formative evaluation design to identify use-related problems and interface improvement needs of a prototype AI-based gait analysis system (MediStep M). The evaluation followed the usability engineering framework described in IEC 62366-1:2020, focusing on user-system interaction and workflow alignment.

### Materials

**Figure 1:**
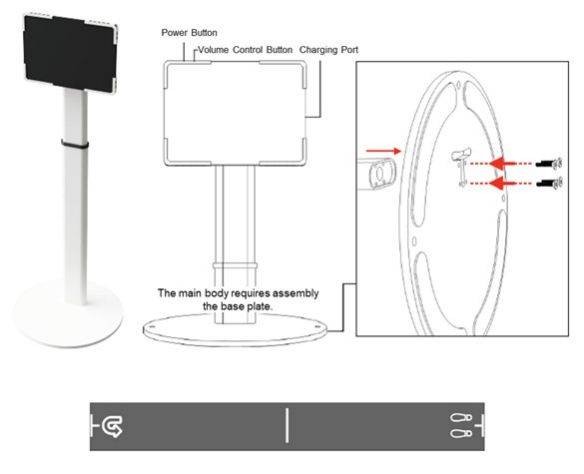
Artificial intelligence–based gait analysis system hardware: tablet and gait mat.

**Figure 2:**
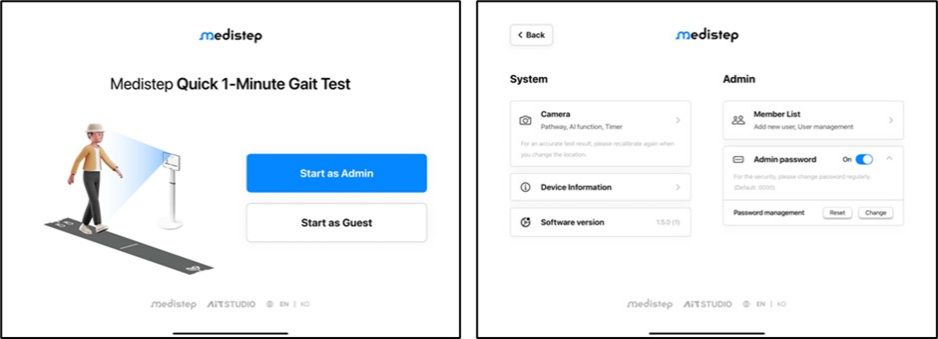
Artificial intelligence–based gait analysis system software interface (start user interface and settings user interface).

MediStep M is a prototype AI-based gait analysis system commissioned by the manufacturer for formative evaluation. The system was designed to enable objective assessment of gait deterioration in older adults and to monitor functional recovery following therapeutic interventions. Currently, in the prototype stage, the system consists of general-purpose hardware (a tablet device) running the MediStep M software (Figures 1-2).

Using the tablet’s built-in camera, the system captures side-view videos of participants performing back-and-forth walking tasks. Through markerless pose estimation technology, the software automatically extracts full-body joint coordinates and computes spatiotemporal gait parameters. These parameters are visualized and quantified to generate comprehensive gait reports, including stride length, walking speed, arm swing amplitude, upper-body inclination, and gait asymmetry. The generated reports are intended to assist clinicians in evaluating gait characteristics and identifying deviations from normative patterns.

### Participant Recruitment

Five licensed physical therapists participated in the study. All participants had a minimum of 5 years of professional experience in gait assessment and rehabilitation. Recruitment was conducted through the National Rehabilitation Center via an internal call for participation.

The inclusion criteria were as follows: (1) licensed physical therapists with clinical experience in gait analysis or rehabilitation, (2) current engagement in gait-related clinical practice, and (3) prior exposure to gait analysis systems or equivalent medical devices.

Participants who were not actively involved in patient treatment or who performed administrative duties only were excluded. The final sample consisted of 4 male therapists and 1 female therapist, reflecting diverse experience levels across inpatient and outpatient rehabilitation contexts.

### Procedures

#### Formative Evaluation Procedures

The formative evaluation was conducted at the National Rehabilitation Center in Seoul, Republic of Korea. Each session was conducted in a controlled clinical environment and lasted approximately 90 minutes (Table 1).

**Table 1:** Formative evaluation procedures, involving practitioners and evaluators, conducted for 90 minutes under the guidance of a facilitator.

Composition	Details	Time (min)
Orientation	The moderator introduced the focus group interview to the participants (physical therapists) and provided an explanation of the product to be discussed, including its purpose, an overview, and the methods involved.	10
Guidance on consent	The moderator informed participants about the video and audio recording procedures. After confirming that the physical therapists fully understood the details of the study, written informed consent was obtained voluntarily. Prior to the interview, the moderator conducted a survey to collect general demographic information from the participants.	10
Perform focus group interview	The moderator conducted user training, demonstrations, and interviews according to a predetermined script. The participants (physical therapists) observed the demonstrations, responded to interview questions, and were allowed to operate the product if necessary. An observer documented the entire interview process.	50
Perform SUS^a^	Following the focus group interview, SUS was administered, followed by an additional interview to gather further feedback from the participants.	20

^a^SUS: system usability scale.

The evaluation consisted of 3 main stages:

1. Product demonstration: The facilitator conducted a live demonstration of MediStep M according to predefined clinical use scenarios. This included device setup, calibration, gait data acquisition, and report interpretation. The demonstration followed the manufacturer’s standard workflow and was intended to simulate typical clinical usage conditions.
2. Participant interaction and observation: Participants observed the demonstration, and when appropriate, were given the opportunity to interact with the system interface (eg, initiating data capture, reviewing gait reports) to better understand its functionality. During this phase, the facilitator encouraged participants to verbalize their thoughts and perceptions regarding usability issues and potential improvements.
3. FGI and SUS assessment: After the demonstration, participants took part in a 60-minute FGI to provide detailed feedback on user interface intuitiveness, workflow integration, and areas for improvement. Subsequently, SUS was administered to quantify perceived usability. Reverse-scored items (2, 4, 6, and 8) were appropriately adjusted before calculating individual and mean SUS scores.

#### Task Scenarios

Table 2 shows the different scenarios in product demonstration, including device setup, calibration, gait data acquisition, and report interpretation.

**Table 2:** Task scenarios.

	Task	Subtask
1	Powering on the device	Connect the charging port and check whether the indicator light turns on. Press the power button located at the upper left on the front panel to turn on the device.
2	Camera calibration	Open the calibration settings window and perform the camera calibration before starting. While monitoring the screen, position the device so that the entire walking path is centered within the camera’s field of view. Adjust the device’s position and orientation to ensure the vertical guideline aligns with the centerline of the walking mat. Resize the guidelines so that both ends of the horizontal guideline align with the T-marks at either end of the walking path.
3	Using the MediStep application	Click the “Start as Administrator” button. Enter the user information into the designated fields on the software interface. Review the displayed information, and then click the “Start AI^a^ Gait Assessment” button.
4	Gait analysis	Select the recording standby timer. Touch the record button.
5	Interpretation of results	The gait analysis results and corresponding gait videos can be reviewed through the report screen.
6	Member management	Adding/deleting members In the member list under the settings menu or on the examiner selection screen, tap the “Add New Member” button. Enter the member’s name, the first 6 digits and the first digit of the last 7 digits of their resident registration number, and their height. Then tap the “Add” button. To delete a member, select the member from the list. A pop-up window will appear with options to edit or delete the information. Tap the “Delete” button. Editing member information This function is accessible only via the member list in the settings menu. Select the desired member; a pop-up window will appear with options to edit or delete their information. After making the necessary changes, tap the “Complete Edit” button.

^a^AI: artificial intelligence.

### FGI Method

**Figure 3:**
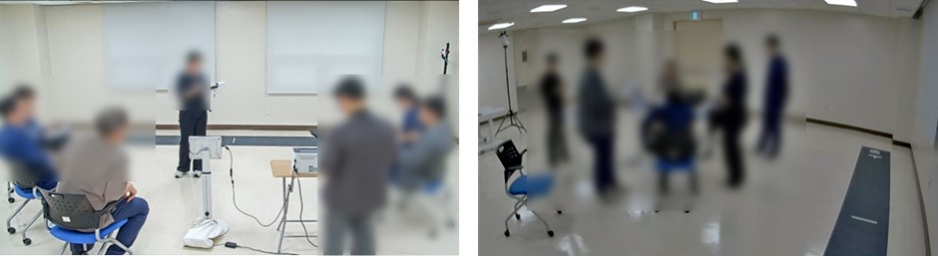
Conducting the focus group interview session.

An FGI is a qualitative research method that collects data through in-depth discussions with intentionally selected participants on a specific topic [16,17]. This approach encourages individuals to openly share their experiences and engage with others’ insights, thereby providing diverse perspectives and rich, detailed information on the subject matter [18]. FGIs are considered an appropriate research method for analyzing and identifying areas for improvement in gait analysis software interfaces from the perspective of clinicians with experience in using similar medical devices (Figure 3). The FGI questionnaire (Table 3) was structured based on the type of questions, including introductory, transitional, key, and closing questions [17].

**Table 3:** Focus group interview questionnaire.

Stage	Questions
Opening questions	General characteristics: (eg, age, gender, clinical experience, experience with similar medical devices) Interview question: Are you currently using gait analysis products or similar medical devices to assess or treat patients?
Transition questions	Could you describe your experience using similar products or medical devices? What types of assessments or treatments have you performed using gait analysis products or similar medical devices? Please include the purpose and methods of the assessments or treatments. In your opinion, what are the advantages of using gait analysis products or similar medical devices for clinical assessments? What key aspects should be considered in the development of gait analysis software?
Key questions	Based on the user manual provided, do you have any comments on the product’s composition (eg, contents, structure, or layout)? Following the verbal explanation and demonstration, do you anticipate any user interface errors or potential hazards when using the MediStep M interface? Are there any elements of the user interface that you believe should be improved? (demonstration provided according to the product scenario.) Do you have any additional comments or suggestions regarding this product?
Ending questions	If you have any further opinions on aspects to consider in the development of gait analysis software, please share them.

### SUS Evaluation Tool

The SUS is a quantitative evaluation tool used to assess how users perceive system usability [19]. The SUS comprises 10 items rated on a Likert scale (1=strongly disagree to 5=strongly agree) (Table 4). The total score was converted to a range of 0-100 points to evaluate overall system usability. Moreover, the SUS scores were assigned a letter grade from “F” (0-60 points) to “A” (91-100 points) according to a standardized grading system [20,21].

**Table 4:** System usability scale questionnaire.

Items	Statement
Utility	I think that I would like to use this system frequently.
Complexity	I found the system unnecessarily complex.
Simplicity	I thought the system was easy to use.
Professionalism (technician support)	I think that I would need the support of a technical person to be able to use this system.
Integration	I found the various functions in the system were well integrated.
Unity	I thought there was too much inconsistency in this system.
Learnability	I would imagine that most people would learn to use this system very quickly.
Convenience	I found the system very cumbersome to use.
Satisfaction	I felt very confident using the system.
Professionalism (prior learning)	I needed to learn a lot of things before I could get going with this system.

### Data Analysis

Qualitative data from FGIs were transcribed verbatim and analyzed using an inductive thematic analysis approach. Two independent researchers coded the transcripts to identify recurring patterns, usability barriers, and improvement suggestions. Themes were derived through iterative comparison and consensus discussions.

Quantitative data from SUS were analyzed descriptively to calculate mean scores, standard deviations, and percentile rankings based on established SUS benchmarks. Both qualitative and quantitative findings were triangulated to derive comprehensive insights into the system’s usability and user needs.

### Ethical Considerations

The study protocol was reviewed and approved by the institutional review board of the National Rehabilitation Center (government-affiliated national rehabilitation center in Seoul, Republic of Korea; NRC-2024-06-046). All participants provided written informed consent prior to participation and were informed that participation was voluntary and that they could withdraw from the study at any time without any penalty or disadvantage. Participants received a nonmonetary gift with a value of up to KRW 50,000 (US $34.05) as a token of appreciation for their participation. To ensure confidentiality, all identifiable personal data were removed, and all photographic materials were anonymized prior to analysis and publication.

## Results

### Participant Characteristics

Five licensed physical therapists participated in the evaluation, with clinical experience ranging from 7 to 23 years (Table 5). All participants had prior experience using similar gait analysis systems (eg, Zebris, Joynt, or Morning Walk) and reported a usage frequency of 1-3 times per week.

**Table 5:** General characteristics of the participants.

ID	Sex	Experience	Similar medical device			
			Used before	Model name	Experience of use	Frequency of use
						
P1	Female	8 y 8 mo	Yes	Zebris	1 y	1 time/wk
P2	Male	18 y 9 mo	Yes	Zebris	1 y	3 times/wk
P3	Male	23 y	Yes	Joynt	1 y	1 time/wk
P4	Male	7 y 8 mo	Yes	Morning Walk	2 y 2 mo	3 times/wk
P5	Male	15 y 7 mo	Yes	Zebris	1 y	2-3 times/mo

### Qualitative Findings (FGI)

FGIs revealed several key themes regarding usability, functionality, and areas for improvement of the AI-based gait analysis system.

#### Theme 1: Importance of Real-Time, Quantitative, and Understandable Feedback

The participants (P1-P5; as indicated in Table 5) appreciated the ability of gait analysis devices to provide quantitative assessments, particularly for aspects that are difficult to measure visually, such as gait symmetry and alignment. They emphasized that real-time feedback not only enhances clinicians' evaluation capabilities but also motivates patients by enabling them to monitor their own gait patterns. However, some participants noted that complex setup procedures and spatial constraints could limit the practical utility of such devices in busy clinical environments.

…The advantage is that machines can accurately measure what we cannot visually quantify, such as distance and symmetry, without requiring large spaces.

P1

…Real-time visualization of movements could help patients self-correct during therapy sessions.

P2

#### Theme 2: Necessity for Simplified and Portable Hardware Design

Although the hardware components and instructions were generally understandable, several participants stressed the need for wireless operations to enhance portability and convenience. The participants recommended minimizing wired connections, incorporating a wireless printer, and adding basic features such as a built-in level gauge for easier setup on uneven surfaces.

…The product should be portable. Wireless operation would greatly improve usability.

P2

…Adding a leveling tool on the device would help ensure proper setup on uneven floors.

P3

#### Theme 3: Challenges Related to Power Button and Battery Display

Many participants expressed frustration with the placement of the power button, which was obscured by the device frame. They suggested relocating it for easier access and improving the visibility of the battery status, either through a larger indicator light or by displaying the battery percentage on the screen.

…The power button is hidden behind the frame, making it very inconvenient to press.

P1

…It would be helpful to see the battery level on the main screen.

P2

#### Theme 4: Calibration Process Needs Automation

The participants criticized the manual calibration process, noting that it could introduce human error depending on the user’s expertise and environmental factors. They recommended an automated calibration to enhance the reliability and usability of the system.

…Manual calibration can vary the results based on a user’s skill. AI-based automatic calibration would significantly increase reliability.

P1

…It should not rely on the inspector’s height but rather on more objective standards like leg length.

P2

#### Theme 5: User Interface and Patient Management System Improvements

Participants generally found the software interface to be user-friendly but identified specific areas for improvement. They requested enhancements to the patient management system, such as the introduction of an auto-complete search, ID-based patient identification, and simplified patient registration procedures.

…When selecting patients, it’s inconvenient to search by full name. An auto-complete or list view would help.

P3

…It would be better if patients were identified by number rather than name for privacy and speed.

P5

#### Theme 6: Enhancing the Gait Analysis Reporting and Interpretation

Participants appreciated the effort to make the results accessible but criticized the oversimplification of the analysis outputs. The authors called for more detailed and clinically meaningful metrics and stronger reference explanations, particularly for cases involving pathological gait patterns.

…Simple statements like slow walking speed aren’t enough. Specific advice is required, such as suggesting an increase in stride length by 10 cm.

P4

…The report distinguishes between normal and pathological gait patterns.

P1

#### Theme 7: Additional Requirements for Clinical Usability

Finally, the participants highlighted the need for expanded language support for international use, improved data extraction features (eg, exporting to Excel), and improved integration with hospital electronic medical record (EMR) systems. Some participants suggested adding remote-start features to minimize device manipulation during testing and prevent device tilting.

…If the device is intended for hospital use, data should be easily exportable and integrated with the EMR.

P3

…Remote control start could prevent tilting issues during test initiation.

P2

### Quantitative Findings (SUS)

**Figure 4:**
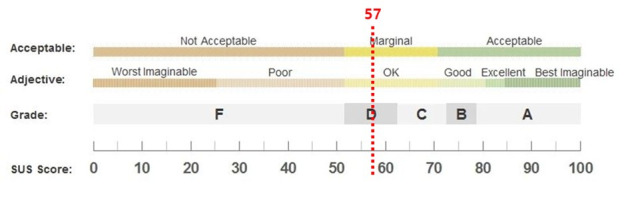
System usability scale (SUS) results.

The mean SUS score was 57, corresponding to a grade of “D” on the SUS grading scale (Figure 4, Table 6).

**Table 6:** System usability scale evaluation results (N=5).

Item	Min (point)	Max (point)	Mean (SD)	Scaled mean (SD)
Total	—^a^	—	2.88 (0.44)	57.00 (17.98)
Utility (Q1)	1.00	3.00	2.40 (0.89)	35.00 (20.00)
Complexity (Q2)	1.00	3.00	2.00 (0.71)	75.00 (15.81)
Simplicity (Q3)	4.00	5.00	4.20 (0.45)	80.00 (10.00)
Professionalism (technician support) (Q4)	2.00	4.00	3.40 (0.89)	40.00 (20.00)
Integration (Q5)	1.00	3.00	2.00 (0.71)	25.00 (15.81)
Unity (Q6)	1.00	4.00	3.20 (1.30)	45.00 (29.15)
Learnability (Q7)	3.00	5.00	4.00 (0.71)	75.00 (15.81)
Convenience (Q8)	1.00	4.00	2.20 (1.10)	70.00 (24.49)
Satisfaction (Q9)	3.00	4.00	3.20 (0.45)	55.00 (10.00)
Professionalism (prior learning) (Q10)	1.00	3.00	2.20 (0.84)	70.00 (18.71)

^a^Not applicable.

## Discussion

### Principal Findings

This formative usability evaluation identified key strengths and limitations of the prototype AI-based gait analysis system MediStep M from the perspective of clinical end users. Overall, the findings demonstrate that while the system shows strong potential to support objective gait assessment in rehabilitation, several usability issues must be addressed to enable efficient integration into clinical practice. The mean SUS score of 57 (grade D) indicates below-average usability, aligning with qualitative feedback obtained through FGIs.

Participants acknowledged the clinical value of quantitative gait analysis enabled by AI-based systems, particularly the ability to objectively visualize spatiotemporal gait parameters that are difficult to assess through observation alone. However, multiple usability barriers were identified across both hardware and software components. Hardware-related issues included limited portability, poor accessibility of the power button, and lack of a battery status indicator. Software-related challenges involved the need for automated calibration, improved patient management features, and more detailed gait reporting to support clinical decision-making. These findings highlight that usability and clinical applicability depend not only on algorithmic performance but also on the intuitiveness and efficiency of the overall user interface and workflow design.

The results of this study are consistent with prior usability research on AI-driven and markerless gait analysis systems, which similarly reported barriers such as setup complexity, calibration burden, and limited interoperability with EMR systems. Previous studies have also emphasized that successful implementation of AI technologies in rehabilitation requires a user-centered approach that reflects real-world clinical workflows [22-25]. Compared to treadmill-based systems such as Zebris, MediStep M offers an overground and portable configuration that allows a more natural gait pattern to be analyzed. Nonetheless, iterative refinement remains necessary to enhance user experience, workflow compatibility, and data management efficiency.

The findings from this study suggest several priorities for future development. First, hardware design should improve ergonomics and mobility through compact structure, wireless operation, and automated leveling mechanisms. Second, calibration procedures should be automated using AI-assisted alignment algorithms to reduce operator variability. Third, software feedback should evolve beyond raw numerical data to include graphical trends, interpretive summaries, and actionable recommendations for rehabilitation. Finally, interoperability features such as secure EMR integration and standardized data export formats are essential for broader clinical adoption.

Importantly, several of the usability issues identified in this formative evaluation were subsequently addressed by the manufacturer. As of March 2025, the finalized version of MediStep M incorporated significant hardware and software refinements, including a redesigned rear magnetic detachable cover, removal of the front frame obstructing the power button, and addition of a main-screen battery status indicator. Wireless printing capability using Apple AirPrint, an AI-based automated calibration algorithm achieving 97% accuracy compared to the manual process, and a CSV-based data export function were also implemented. This iterative process underscores how formative usability evaluations can directly inform product improvement and regulatory readiness.

### Limitations

Several limitations should be acknowledged. The small sample size (N=5) limits the generalizability of our findings, and the predominance of male participants (4 out of 5) may introduce gender bias. As a formative study, the evaluation did not measure long-term usability or clinical efficacy outcomes. Future studies should include larger and more heterogeneous samples, repeated testing cycles, and longitudinal assessments to validate the system’s usability and clinical performance after iterative refinement.

### Conclusion

This study provides evidence on the usability of an AI-based gait analysis system from the perspective of clinical end users. Although the prototype demonstrated technical feasibility and clinical potential, several usability challenges were identified that require further optimization. The subsequent improvements implemented by the manufacturer exemplify how formative feedback can guide user-centered development of rehabilitation technologies, supporting safer and more effective integration of AI systems into clinical workflows.

## Data Availability

The datasets generated and/or analyzed in this study are available from the corresponding author upon reasonable request.

## Abbreviations

AI: artificial intelligence
EMR: electronic medical record
FGI: focus group interview
SUS: system usability scale
